# Ablation of atrial fibrillation with the Epicor system: a prospective observational trial to evaluate safety and efficacy and predictors of success

**DOI:** 10.1186/1749-8090-5-34

**Published:** 2010-05-05

**Authors:** Simon Schopka, Christof Schmid, Andreas Keyser, Ariane Kortner, Julia Tafelmeier, Claudius Diez, Leopold Rupprecht, Michael Hilker

**Affiliations:** 1 Department of Cardiothoracic Surgery, University Medical Center Regensburg, Germany

## Abstract

**Background:**

High intensity focused ultrasound (HIFU) energy has evolved as a new surgical tool to treat atrial fibrillation (AF). We evaluated safety and efficacy of AF ablation with HIFU and analyzed predictors of success in a prospective clinical study.

**Methods:**

From January 2007 to June 2008, 110 patients with AF and concomitant open heart surgery were enrolled into the study. Main underlying heart diseases were aortic valve disease (50%), ischemic heart disease (48%), and mitral valve disease (18%). AF was paroxysmal in 29%, persistent in 31%, and long standing persistent in 40% of patients, lasting for 1 to 240 months (mean 24 months). Mean left atrial diameter was 50 ± 7 mm. Each patient underwent left atrial ablation with the Epicor system prior to open heart surgery. After surgery, the patients were treated with amiodarone and coumadin for 6 months. Follow-up studies including resting ECG, 24 h Holter ECG, and echocardiography were obtained at 6 and 12 months.

**Results:**

All patients had successful application of the system on the beating heart prior to initiation of extracorporeal circulation. On average, 11 ± 1 ultrasound transducer elements were used to create the box lesion. The hand-held probe for additional linear lesions was employed in 83 cases. No device-related deaths occurred. Postoperative pacemaker insertion was necessary in 4 patients. At 6 months, 62% of patients presented with sinus rhythm. No significant changes were noted at 12 months. Type of AF and a left atrial diameter > 50 mm were predictors for failure of AF ablation.

**Conclusion:**

AF ablation with the Epicor system as a concomitant procedure during open heart surgery is safe and acceptably effective. Our overall conversion rate was lower than in previously published reports, which may be related to the lower proportion of isolated mitral valve disease in our study population. Left atrial size may be useful to determine patients who are most likely to benefit from the procedure.

## Background

Atrial fibrillation (AF) is the most commonly sustained cardiac rhythm disorder. An estimated 4.5 million people in the European Union and 2.2 million people in North America suffer from paroxysmal or persistent AF [1]. All types of AF are associated with a varying increased risk of stroke and heart failure, and the overall mortality rate is doubled as compared to people with normal sinus rhythm [2].

In cardiac surgery, the percentage of patients with concomitant AF is increasing because AF is linked to structural heart disease and the number of patients with markedly advanced heart disease is growing. AF has also been identified as an important risk factor for both mortality and morbidity in cardiac surgery [3]. Recent studies have shown that 10-year survival of patients undergoing coronary bypass surgery without conversion of atrial fibrillation into sinus rhythm is reduced by 24% [4].

**Figure 1:**
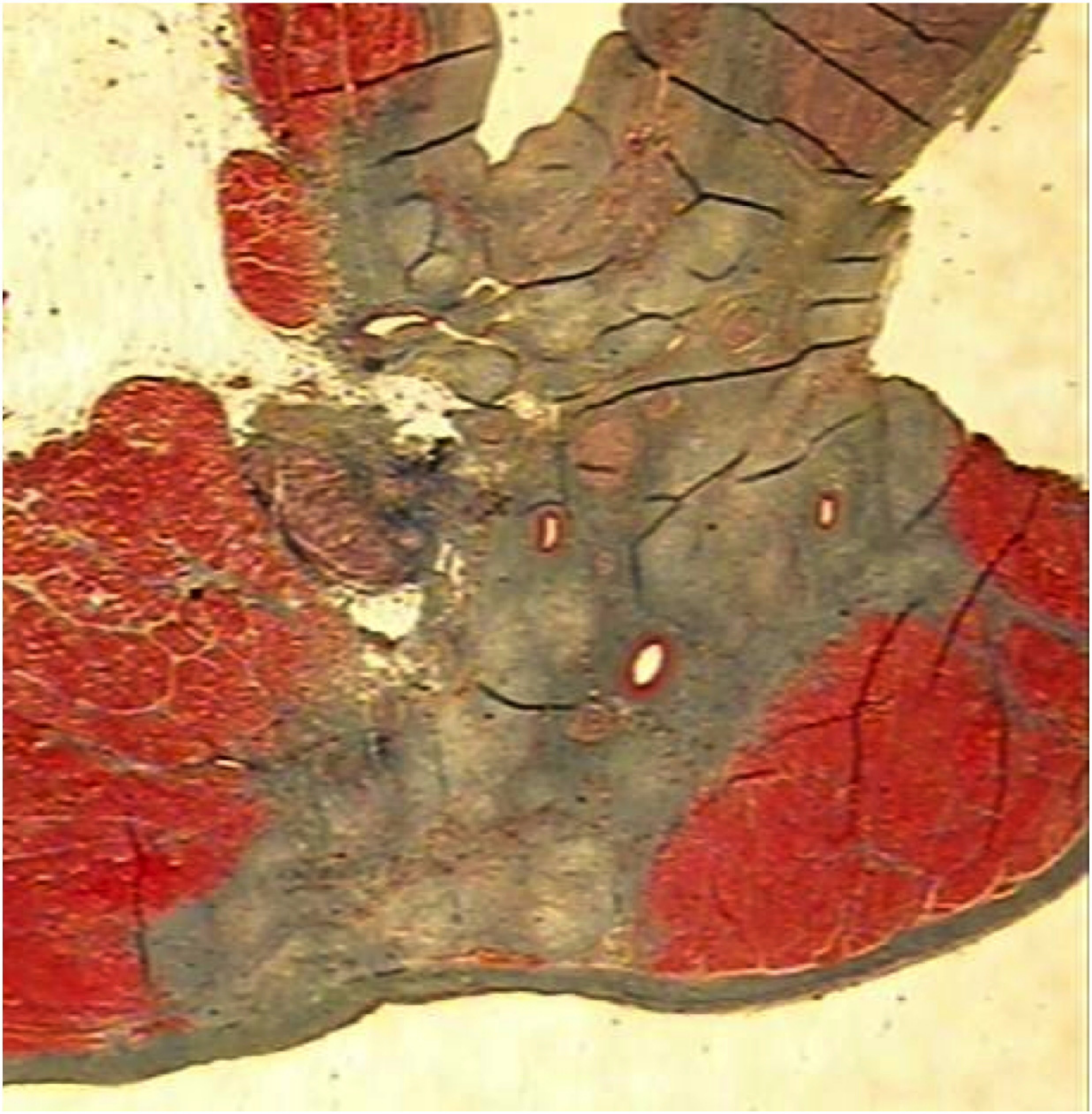
**Ultrasound ablated bovine myocardium**. The scar shows a transmural lesion with the coronary vessels in the ablated section remaining patent. With permission of St. Jude Medical.

Surgical treatment of AF began in 1987 with the Maze procedure developed by Cox. Although the procedure resulted in freedom from AF in 80% to 95% of patients after 15 years of follow-up, it was never widely accepted because of its complexity and invasiveness [5]. Instead, less extensive alternative treatment modalities utilizing different energy sources for epi- or endocardial application, such as radiofrequency, laser, microwave and cryotherapy, were devised to avoid the cut-and-sew technique and to simplify surgery by creating a so-called box lesion or pulmonary vein isolation [6]. Clinical studies analyzing success rates of devices using these energy sources detect success rates ranging from 65% to 90%, depending on the energy source as well as patient characteristics and follow up [6]. A particular problem of these new ablation techniques is to create transmural lesions. Transmurality is considered essential but virtually impossible to control. Accordingly, an exaggerated use of endocardial radiofrequency application has lead to damage at surrounding structures, especially to esophageal injuries [7]. Epicardial employment of high energy involves problems with regard to the heat sink effect and energy propagation through epicardial fat, and thus may also damage surrounding structures [8]. In contrast, AF ablation with high intensity focused ultrasound (HIFU) creates transmural lesions without the need of large thermal gradients and without jeopardizing adjacent structures (Figure 1) [6].

The purpose of this study was to evaluate the efficacy of left atrial HIFU ablation of AF with the Epicor Cardiac Ablation System (St. Jude Medical, Maple Grove, MN) as concomitant procedure during open heart surgery and to assess predictors of success.

## Materials and methods

### Patients

From January 2007 to June 2008, 110 consecutive patients scheduled for first-time open heart surgery and known AF were prospectively enrolled into the study, which was approved by the local ethic committee. Written informed consent was obtained from all patients. The study cohort included 66 men and 44 women with a mean age of 71.2 years (Table 1). Primary diagnosis was ischemic heart disease in 48%, aortic valve disease in 50%, and mitral valve disease in 18% of patients. Twenty-five percent of patients required combined cardiac surgery (Table 2). Patients with ischemic heart disease were diagnosed with paroxysmal AF in 38%, with persistent AF in 35%, with longstanding persistent AF in 26% of cases, for patients with aortic heart disease the percentage for the different types of AF were 21% paroxysmal, 34% persistent, 43% long standing persistent. Accordingly patients undergoing combined cardiac surgery suffered in 20% of cases of paroxysmal, in 37% of persistent, and in 41% of cases of long standing persistent AF. There was no statistical significant correlation in between type of AF and diagnosis group. Preoperative left ventricular ejection fraction was 54.8 ± 9.6%, preoperative left atrial diameter was 50.2 ± 6.9 mm on average.

**Table 1:** Preoperative data of patients enrolled into the study.

Variable	Mean	SD	Range
Age in years	71.8	7.5	48-86

AF duration in months	36.4	46.7	1-240

LA size	50.3	6.9	30 - 68

LVEF	54.8	9.7	27 - 70

EuroSCORE	7.6	6.6	0.9-43.9

**Table 2:** Procedures concomitantly conducted to left atrial epicardial ablation, absolute numbers and percentage of total

Procedure	Number	Percentage of Total
Isolated aortic valve repair/replacement	30	27%
Aortic valve + CABG	12	

Isolated mitral valve repair/replacement	13	11%
Mitral valve + CABG	3	

Isolated CABG	37	33%

Double valve surgery	12	10%
Tricuspid valve reconstruction	6	
ASD closure	2	

Ascending aorta replacement	3	

Combined cardiac surgery	27	25%

Diagnosis and classification of AF were established by standard ECG and holter ECG in all patients prior to admission. AF was defined paroxysmal in 29%, persistent in 31% and long standing persistent in 40% of patients. According to the Cox classification, AF was continuous in 71% and intermittent in 29% of patients. Atrial diameters were significantly smaller in patients with intermittent AF than in patients with continuous AF. 16 patients underwent an ineffective attempt of rhythm control prior to admission.

### Surgical technique

In all patients, ablation of AF was performed via a midline sternotomy prior to the initiation of extracorporeal circulation for the subsequent cardiosurgical procedure.

The Epicor Positioning and Sizing (PAS) System, which was designed to indicate the proper UltraCinch device size and act as a guide for simple placement of the device was passed behind the superior vena cava through the transverse sinus and the oblique sinus underneath the inferior vena cava. After measurement of the proper size, the sizer was replaced by the UltraCinch ablation device to create a "box" lesion above the ostia of all four pulmonary veins. The two ends of the UltraCinch were approximated with tourniquets to snug the device securely around the left atrium. After flushing of the transducer elements with saline solution the ablation cycles were initiated and automatically progressed by the Epicor Ablation Control System until completion. During the ablation process, that takes about 10 minutes, the surgical preparations for extracorporeal circulation were continued. According to the surgeon's preference additional linear lesions were created with the UltraWand, particularly, a mitral line that extends from the "box" lesion to the mitral valve annulus.

### Postoperative protocol

After surgery, all patients were treated with intravenous heparin and amiodarone. With reconvalescence of the patient, anticoagulation (warfarin or coumadin) and antiarrhythmic therapy (amiodarone 200 mg/d) were switched to oral medication and maintained for 6 months. After 6 months continuance of oral anticoagulation was determined on basis of the CHADS_2 _score. In case of contraindications for amiodarone treatment, the latter was replaced by a beta blocker, preferably sotalol. If AF persisted or recurred external cardioversion/defibrillation was not recommended in the early postoperative period to allow full maturation of the ablation line, which takes up to three month in lesions created by ultrasound. After 6 months Amiodarone was discontinued and replaced by β-blockage.

A physical examination, standard 12-lead ECG, 24-hour Holter monitoring, and transthoracic echocardiography were conducted systematically at 6 and 12 month follow-up visits.

### Statistical analysis

Data were entered into a computerized database and analyzed with a statistical package (STATISTICA; StatSoft, Inc). The descriptive summary of data included mean ± standard deviation and 95% confidence intervals for continuous variables and proportions for categorical variables. Between-group differences were assessed with t-tests and analysis of variance in more than two groups for continuous variables and Fishers exact tests for nominal variables. The significance of variables on the odds of procedure failure was assessed by univariate logistic regression analyses. All reported p-values are two-sided.

## Results

### Safety evaluation

All patients had successful application of the system on the beating heart prior to initiation of extracorporeal circulation. On average, 11 ± 1 ultrasound transducer elements were used to create the box lesion. The hand-held probe for additional linear lesions was employed in 83 cases. No device-related or procedure-related complications occurred, particularly no injury of the esophagus, coronary arteries or, phrenic nerve. Within the 30-day period, non-lethal postoperative complications including temporary neurological dysfunction (cognitive disorder) in 6 patients and mild pneumonia in 7 patients were observed. Insertion of a long standing persistent pacemaker was necessary in 4 patients who underwent aortic valve replacement, combined with coronary bypass surgery in 3 patients, and isolated in 1 patient. One patient experienced an episode of atrial flutter 3 months after surgery. and underwent successful interventional ablation with subsequent stable sinus rhythm.

In-hospital, mortality was 3.6%. Two patients each died of mediastinitis and of mesenterial ischemia and retroperitoneal bleeding, respectively. Three out of the 4 patients were > 70 years. The EuroSCORE of these patients was 19.9 on average.

### Evaluation of efficacy

All patients were alive at follow-up, complete data on cardiac rhythm were obtained in 98% of cases. Mean follow-up interval was 7.8 months, with 75 patients followed for 6 months and 49 patients for 12 months.

Overall freedom from AF, i.e. stable sinus rhythm, was noted in 62% of patients at 6 months and 65% of patients at 12 months after surgery, which was not significantly different. Postoperative pacemaker stimulation for bradycardia remained necessary in 4 patients.

**Figure 2:**
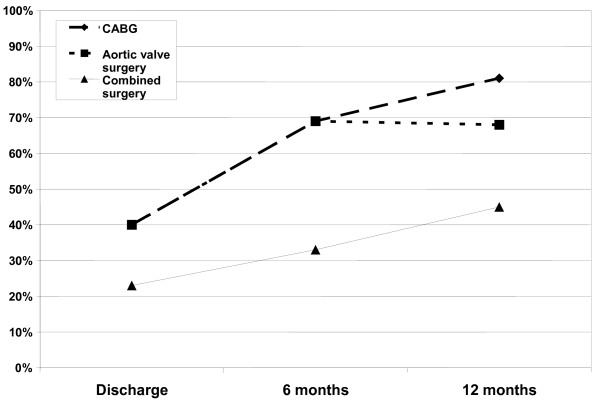
**Patients with sinus rhythm after undergoing CABG, aortic valve disease and combined cardiac surgery at discharge, 6 and 12 months follow-up**.

The influence of the concomitantly performed cardiac procedure on the success rate of AF ablation of is depicted in figure 2. Best results were obtained in patients undergoing coronary artery bypass surgery (CABG), who presented with 69% freedom from AF at 6 months and a 81% freedom from AF at 12 months follow-up (p = 0.47). Patients with aortic valve replacement demonstrated a comparable outcome with 69% and 68% freedom from AF at 6 and 12 months, respectively (p = 1.0). The patient population undergoing isolated mitral valve surgery was too small for a valid comparison. Combined cardiac surgery was associated with a much lower success rate, with only 33% and 45% freedom from AF at 6 and 12 months, respectively (p = 0.69). In these patients, a larger (preoperative) left atrial diameter was noted (as was also seen in the mitral valve patients) compared to patients with isolated ischemic heart disease or aortic valve disease (p = 0.019).

**Figure 3:**
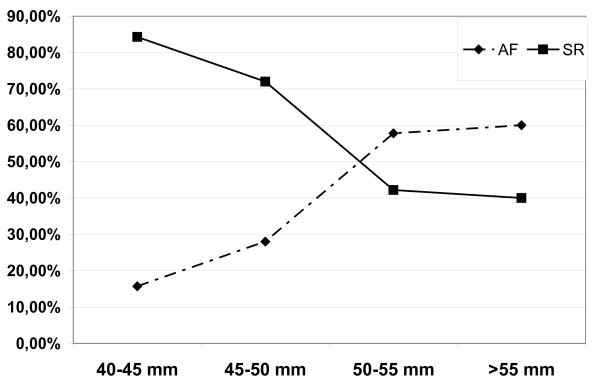
**Percentage of patients with sinus rhythm or atrial fibrillation after 12 months follow-up dependent on preoperative left atrial size**.

**Figure 4:**
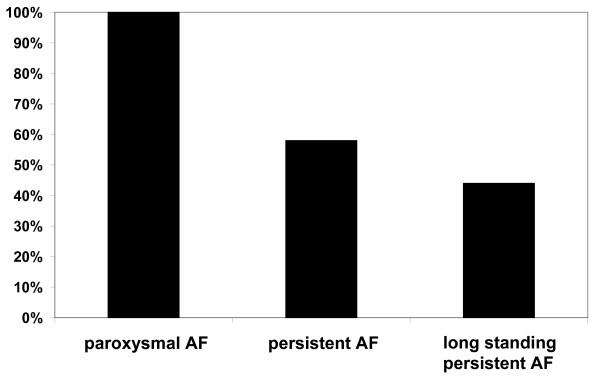
**Preoperative atrial fibrillation and percentage of patients with sinus rhythm postoperatively**. Paroxysmal atrial fibrillation differs significantly from both persistent and long standing persistent atrial fibrillation, whereas no difference exists between persistent and long standing persistent atrial fibrillation.

Preoperatively assessed parameters including type of AF, duration of AF, left atrial diameter, left ventricular ejection fraction as well as the size of the UltraCinch ablation device (number of transducer elements) were evaluated as predictors of success. The type of AF had a profound impact on the success rate. At 12 months, sinus rhythm was obtained in 100% of patients with paroxysmal AF, in 58% of patients with persistent AF, and in 44% of patients with long standing persistent AF (Figure 3). The left atrial diameter measured by echocardiography was highly predictive too. A cut-off point was seen at a diameter of 50 mm (m-mode, parasternal long axis). Patients with a left atrial diameter < 50 mm presented sinus rhythm in 77% of cases, in contrast to patients with a left atrial diameter > 50 mm, who had only a success rate of 41% (p = 0.0015) (Figure 4). Accordingly, a left atrial diameter > 50 mm resulted in an odds-ratio of 4.2 (95% confidence interval 1.88 to 12.64). Preoperative left ventricular ejection fraction, size of the UltraCinch ablation device, and duration of AF prior to surgery did not reveal predictive value.

## Discussion

AF as a common co-morbidity in cardiac surgical patients with a prevalence of AF in about 10% of patients with ischemic heart or aortic disease, and in almost 50% of patients with mitral valve disease [9,10]. As uncorrected AF is associated with increased morbidity and mortality in patients undergoing cardiac surgery, AF ablation concomitant to cardiac surgery appears to be an efficient measure to improve outcome.

The intention of this prospective study was to assess safety of the Epicor ablation procedure and to identify predictors for success and failure, respectively. Our study population was not biased towards a positive selection. In fact, every consecutive patient who wanted to participate was included, which is also expressed by the average age of 71 years and the EuroSCORE of 7.6. In contrast to previous reports, most of our patients suffered from ischemic heart disease and aortic valve stenosis, while only a minority had mitral valve disease. Twenty-five percent of patients underwent combined cardiac surgery. These patients often have rather complex medical histories and significant co-morbidity. If the cardiac surgery is extended by ablation of AF, the latter procedure should add only minimal risk to the patient. Ultrasound ablation is a particularly appropriate intervention for these patients since no bypass or cross clamp time is added to the procedure. In contrast to an endocardial ablation, atriotomy is not required as ultrasound is applied epicardially on the beating heart. Accordingly, an Epicor ablation may also be performed in off-pump CABG and as a stand-alone procedure. A previous report from Ninet et al. already stated an excellent safety of the system, which is consistent with our experience. We did not observe any procedure-related adverse event or postoperative complication, and the need for a postoperative pacemaker implantation was low [6].

An appropriate postoperative management is important after AF surgery. Since the maturation of the transmural lesions and the electrical isolation of arrhythmic foci may take up to 3 months, electrical cardioversion was avoided during this period. Instead, patients were treated with amiodarone to reduce the occurrence of arrhythmia during the early phase after surgery [11,12]. As an evaluation of success is not possible within the first 3 months, the first follow-up visit was scheduled at 6 months after surgery.

The overall freedom from AF with 65% was lower than in previous publications with success rates of about 85% [13,6]. This difference cannot be proven by less efficiency of the ablation system since no interventional electrophysiological studies could be performed during the follow-up visits. However, one may speculate whether the underlying heart diseases did also influence outcome. Our patient cohort mainly suffered from ischemic heart disease and aortic valve stenosis in contrast to previous studies, where mitral valve disease was predominant [6,13]. It is well known, that mere mitral valve surgery (without additional AF ablation) had a significant beneficial effect on conversion to sinus rhythm [14,15]. Accordingly, in prior publications, success rates after ablation of AF were typically superior in patients undergoing mitral valve surgery [6]. A recent study on patients with ischemic heart disease has shown 77% freedom from AF, which is comparable to our results [16].

As has also been shown by others, patients with paroxysmal AF showed a high success rate, significantly better than those with persistent or long standing persistent AF [6,13]. These patients usually suffer from less pathologic alterations of the myocardium including myocyte size, wall thickness, fibrotic changes and left atrial size.

Nevertheless, is should not be forgotten that these good results may have been a result of inadequate diagnostic measures to detect short periods of AF during follow-up exams. Therefore, we now favor the implantation of event recorders in patients who undergo AF ablation.

Our logistic regression analysis presented preoperative LA size as the strongest predictor of the procedure's failure or success, respectively. At a cut-off point of 50 mm failure rate dramatically worsened with an odd ratio of 4.2. The consequences of left atrial enlargement is less well understood, but it has been assumed that the arrhythmogenic foci known to trigger AF are then more likely to reside in the left atrium than in pulmonary veins [17,18]. In the literature, the duration of AF also showed to have a predictive value but this could not be confirmed in our analysis [6,7]. However, the duration of AF was usually obtained via the medical history, which is of limited reliability because of the common occurrence of asymptomatic AF.

The size of the transducer system, i.e. the number of transducer elements, was not predictive too, and did not correlate with echocardiographically measured left atrial size.

In conclusion, we found the ultrasound epicardial ablation of AF with the Epicor system, concomitantly applied with cardiac surgery, to be a safe procedure. Effectiveness of the ablation depends on the primary diagnosis as well as on the preoperative left atrial size. Thus, these variables may serve as a tool to preoperatively identify patients who are most likely to benefit from this procedure. Further analysis of the Epicor registry, a multicenter registry of patients undergoing ultrasound ablation, is going to overcome the major limitation of the present study, i.e. the low number of study participants.

## Limitations of the study

The results of this study are limitated by the inhomogenous study population, which is partly founded in the fact that patients have been recruited consecutively. Another limitation is the duration of follow up. The manuscript presents data of the first 110 patients treated with this device in the center, analysis of a multicenter registry will follow. The most important limitation is the inhomogenous usage of the additional device creating a mitral line, further analysis will focus on regular usage of this device and study its influence on freedom of AF.

## Competing interests

The authors declare that they have no competing interests.

## Authors' contributions

SS carried out follow up's and drafted the manuscript. CS participated in design and coordination of the study and helped to draft the manuscript. AK coordinated the study and helped performing follow up studies. AK performed follow up studies. JT performed follow up studies and helped to draft the manuscript. CD carried out statistical analysis. LR performed surgical ablations. MH conceived of the study, and participated in its design and coordination and helped to draft the manuscript.

All authors read and approved the final manuscript.
